# Performance prediction and stability of maize hybrids in contrasting *Striga* environments

**DOI:** 10.1016/j.jafr.2025.102405

**Published:** 2025-12

**Authors:** Solomon Adeyemi Oyekale, Baffour Badu-Apraku

**Affiliations:** a Pan African University Institute of Life and Earth Sciences (Including Health and Agriculture) (PAULESI), University of Ibadan, Ibadan, Nigeria; b Department of Agriculture, College of Agricultural Sciences, Landmark University, P.M.B, 1001, Omu-Aran, Kwara State, Nigeria; c International Institute of Tropical Agriculture (IITA), P.M.B, 5320, Ibadan, Oyo State, Nigeria

**Keywords:** Grain yield stability, *Striga hermonthica*, Hybrid maize, *Striga* damage score, Plant aspect, Genotype main effect plus genotype by environment biplot analysis

## Abstract

Maize yield and production are significantly hampered by *Striga hermonthica*, causing up to 100 % yield loss under severe infestation in Africa. This research sought to: (i) assess whether extra-early maize hybrids' yield and plant aspect performance under *Striga*-free conditions could respectively predict yield and *Striga* host damage performance in *Striga*-infested environment; (ii) identify *Striga*-resistant/tolerant biofortified hybrid maize and; (iii) identify maize hybrid with stable yield and tolerance/resistance to *Striga* stress. To achieve the above, 150 maize hybrids (obtained from the hybridization of 30 lines using the factorial mating design II) and six checks, were examined in *Striga*-free and *Striga*-infested environments at different locations in Nigeria over two years. The regression coefficients and R^2^ obtained when plant aspect and grain yield across *Striga*-free conditions were used to predict *Striga* host damage and grain yield across *Striga* infestation and vice versa were below 25 % and 4 %, respectively. These indicate that extra-early maize hybrids’ response in one environment did not reliably predict the maize response in the other with respect to the traits concerned. Mean yields of the maize hybrids were 4.9 and 3.3 t/ha in *Striga*-free environment and under *Striga* infestation, respectively. Among the 150 hybrids, LINE 61 × LINE 49 exhibited the highest *Striga* base index value of 13.6, marking it as the most *Striga*-resistant/tolerant hybrid identified in the study. Genotype main effect plus genotype × environment biplot analysis identified LINE 44 × LINE 55 and LINE 61 × LINE 49 as stable and high-yielding hybrids in *Striga*-free and *Striga*-infested conditions, respectively. These hybrids are recommended for on-farm evaluation, and if found superior, should be subsequently released to maize growers in areas affected by *Striga hermonthica* to boost maize production and contribute to food security in Africa.

## Highlights

• *Striga hermonthica* reduced maize productivity by 33 % in this study.
• Maize grain yield under *Striga* infestation could not be predicted from maize grain yield under *Striga*-free environment.
• Plant aspect score is not a reliable predictor of *Striga damage* score – an index of *Striga* tolerance in maize.
• Different maize hybrids were identified as high-yielding and stable in the contrasting *Striga* environments.

## Graphical abstract

**Figure:**
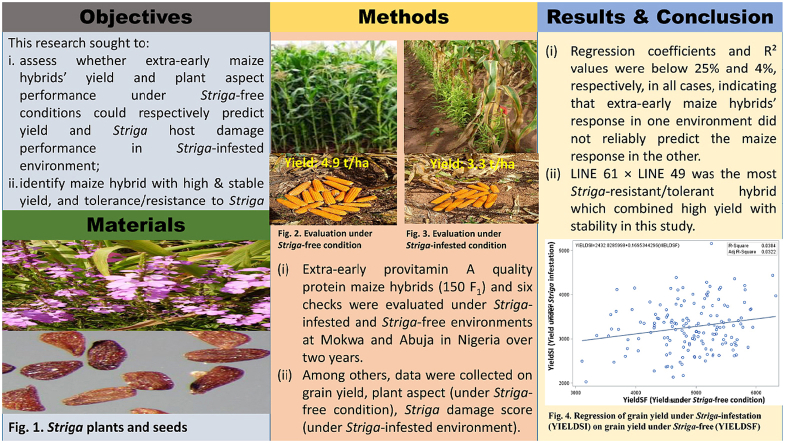

## 1 Introduction

Maize (*Zea mays* L.) is an important member of *Poaceae* family that makes significant contribution to food security in Africa and the world at large. It is usually cultivated for its grains that is rich in calories, vitamins and protein and serves as food for several people in sub-Saharan Africa [1]. However, the average maize yield in Central and West Africa (CWA) countries remains around 2 t/ha [2]. This yield is hampered by biotic stresses, such as *Striga hermonthica* [Del. Benth] [3,4], armyworms [5], and abiotic stresses [[6], [7], [8]]. Among these, biotic stresses significantly affect crop yield and play a crucial role in genotype × environment interactions [9].

*Striga* is a key biotic threat to maize production/yield in the savannas of CWA [10]. Over 30 % of African countries, particularly in Southern, Central, and West Africa, face high *Striga* infestations, which can lead to nearly 100 % crop losses [4,11,12]. Some farmers have even abandoned their fields due to severe *Striga* infestation, while many others struggle with extremely low maize yields. This situation drastically reduces farmers' incomes and hampers maize's contribution to food security, thereby affecting the attainment of the United Nations Sustainable Development Goals of no poverty and zero hunger (Goals 1 and 2, respectively) in WCA.

Various methods have been developed and deployed to control *Striga*, including cultural, biological, the use of germination stimulants, resistant varieties, and chemical methods, among others [[12], [13], [14]]. However, no single method is sufficient for effective control of this holoparasitic and hemiparasitic weed [12]. Thus, integrated *Striga* management (ISM), which combines multiple methods, is advocated. A study by Ref. [15] showed that ISM technology improves maize yield performance. A key component of ISM is the use of maize varieties that are resistant to *Striga* – an approach which is considered the most economical and sustainable [14].

Traditionally, field evaluations to select *Striga*-resistant maize varieties are conducted under both artificial *Striga* infestation and *Striga*-free conditions — a labor-intensive, costly, and time-consuming process. Predicting maize performance based on grain yield and other traits with strong correlations to yield, in either *Striga*-free or *Striga*-infested environments, could reduce the effort, time, and cost of identifying *Striga*-resistant varieties. Moreover, the grain yield stability of a maize genotype across *Striga*-infested environments reflects the plant's inherent potential to tolerate or resist *Striga* stress. Thus, breeding and adopting *Striga*-resistant, biofortified maize hybrids with extra-earliness, high yield, and stability can significantly contribute to nutrition, multiple cropping systems, and ISM in CWA [16,17]. Genotypes with stable performance across years and locations can be identified through multi-environment trials [9]. Stability in a genotype across various environments is a function of its interaction with the environment — a stable genotype has minimal interaction with the environment, while a less stable genotype shows greater interaction [18].

Various statistical methods, including univariate parametric statistics, nonparametric, and graphical stability analyses, have been used to identify stable crop genotypes in different studies [[19], [20], [21], [22]]. However, genotype main effect plus genotype × environment interaction (GGE) biplot analysis is commonly used to study genotype stability in various crops, as it provides graphical information on genotype and mega-environment evaluations [22]. Hence, this study was undertaken to: (i) assess whether grain yield and *Striga* damage scores under artificial *Striga* infestation could be predicted from grain yield and plant aspect under *Striga*-free environments, respectively, and vice versa; (ii) identify *Striga*-resistant/tolerant biofortified hybrid maize which might be suitable for ISM (and as pools of promising alleles for developing superior maize varieties) and; (iii) identify maize hybrids with combined performance for stability and high yield across the research environments.

## 2 Materials and methods

### 2.1 Genetic materials

Thirty fixed maize lines (with the following agronomic and nutritional qualities: extra-earliness, tolerance to low soil nitrogen, *Striga hermonthica* tolerance, provitamin A, tryptophan and lysine) were crossed to develop 150 F_1_ hybrids using the factorial mating design II. Different responses of the inbred lines were used for their categorization as tolerant or susceptible to *Striga* during field evaluations, as identified by IITA's *Striga* base index, coupled with results of screening under light box to identify inbred lines with quality protein maze traits [23]. Twenty-three of the inbred lines expressed tolerance to *Striga* stress while 7 were susceptible (Supplemental Table 1). The six sets of the maize lines were mated using the North Carolina II version [24] to generate 94 tolerant × tolerant, 25 tolerant × susceptible, 24 susceptible × tolerant and 7 susceptible × susceptible F_1_ hybrids (Supplemental Table 2).

### 2.2 Experimental locations and field trials

Evaluations of the 150 F_1_ hybrids with six checks were carried out in contrasting *Striga* environments namely: *Striga*-infested and *Striga*-free environments at Abuja (9°15' N, 7°20' E, 300 m altitude, 1700 mm annual precipitation) and Mokwa (9°18' N, 5°4' E, 457 m altitude, 1100 mm annual precipitation) in 2016 and 2017. Due to constraint of land availability, evaluation of the hybrids was not conducted under *Striga*-free conditions at Abuja in 2016. The locations are in southern Guinea savanna agro-ecological zone of Nigeria, with monomodal rainfall pattern and *Striga* infestation seriously constraining maize production [25]. At each environment, a 12 × 13 alpha-lattice design was used with the hybrids replicated twice. The experimental unit was a single row plot 3 m long. Spacing between rows was 0.75 m while spacing within each row was 0.40 m. Before sowing of maize seeds under *Striga*-infested environment, artificial inoculation of planting holes per plot with viable *Striga* seeds was carried out using well-calibrated scoops [26]. About 9 g of sand-seed mixture was used to infest each seed hole (with about 15, 000 seeds of *Striga* with 33 % viability) prior to sowing of three maize seeds per hole. At 2 weeks after planting (WAP), maize seedlings in every plot were thinned to two plants per hill to give approximately 66, 667 plants/ha. At 2 WAP, the *Striga*-free environment received 45 kg N ha^−1^ (urea), 60 kg P ha^−1^ (single superphosphate) and 60 kg K ha^−1^ (muriate of potash). Urea was later applied at four weeks after planting using 45 kg N ha^−1^. Under *Striga*-infested environment, however, NPK (15-15-15) fertilizer, 30 kg ha^−1^, was applied to supply nitrogen, phosphorus and potassium to the plant in equal proportion. Other weeds, apart from *Striga*, were hand-pulled under *Striga*-infested environment while standard practices were employed under *Striga*-free conditions. Fall armyworms were controlled with ampligo at the rate of 300 ml/ha (active ingredient, 100 g/l chlorantraniliprole + 50 g/l lambda-cyhalothrin).

### 2.3 Recording of data

Data were collected on plants per plot. Days to 50 % anthesis (DAN) was estimated as the number of days from the first day of planting till the day 50 % of the maize plants in each plot had shed their pollen. Days to 50 % silking (DYSK) was determined as the number of days for 50 % of the plants in each plot to emerge silks after planting. Anthesis-silking interval was estimated as the difference between DAN and DYSK. Ear and plant heights were measured from the base of the plant to the node supporting the uppermost ear and from the ground level to the first tassel branch, respectively. Plant aspect (determined under *Striga*-free conditions only) was assessed morphologically on a rating scale of 1–9; where scale 1 referred to excellent plant type while scale 9 depicted poor plant type. Ear aspect was scored on a rating scale of 1–9; where scale 1 referred to ears that were well-filled, uniform, large and clean while scale 9 represented ears that were partially-filled, variable, small and rotten [27]. Ears per plant was obtained by dividing the number of ears harvested per plot by the number of plants at harvest. *Striga* emergence counts and *Striga* damage scores were collected under *Striga*-infested environment, both at 8 and 10 WAP. *Striga* emergence count was obtained by counting the number of emerged *Striga* plants around the maize plants per plot while *Striga* damage score was determined on a rank scale of 1–9; scale 1 implied normal plant growth with no visible symptoms while scale 9 referred to complete scorching of all leaves, leading to premature death or collapse of the host plant and no ear formation [26,28,29]. Maize grain yield (t ha^−1^) was estimated, across each of the two environmental conditions, using the cobs’ field weight with 80 % shelling percentage at 15 % adjusted moisture level.

### 2.4 Statistical analyses

Analysis of variance (ANOVA) was carried out on grain yield and other agronomic traits collected across environments using PROC GLM in statistical analysis system (SAS) version 9.4 [30]. Grain yield data and other variables, common to both environments, were used in the analysis across environments. Before the analysis, *Striga* emergence count, *Striga* damage score, ear aspect and plant aspect were not normally distributed, hence they were log-transformed. Traits with significantly different means were subjected to post-hoc analysis using the least significant difference at the 5 % probability level. Treatment (*Striga*)-location-year represented an environment in the analysis. Hybrid was regarded as a fixed factor while blocks in each replication, replications and environments were regarded as random factors; hence the mixed model was used for the analysis.

Assessment of hybrid performance across the four *Striga*-infested environments was carried out with the IITA's *Striga* base index. The index incorporates the following: *Striga* damage score (an index of *Striga* tolerance), grain yield, *Striga* emergence count (an indicator of *Striga* resistance) and number of ears per plant. The index is used to ascertain whether a hybrid is *Striga*-tolerant or susceptible. The base index is as shown below:

*Striga* base index = 2*MGY* + *EPP* – (*SHD*_*1*_ + *SHD*_*2*_) – 1/2(*SEC*_*1*_ + *SEC*_*2*_),

where, *MGY* = maize grain yield (t/ha) under *Striga*-infested environment, *EPP* = ears per plant, *SHD*_*1*_ = *Striga* host-damage score at 8 WAP, *SHD*_*2*_ = *Striga* host-damage score at 10 WAP, *SEC*_*1*_ = *Striga* emergence count at 8 WAP, *SEC*_*2*_ = *Striga* emergence count at 10 WAP [31].

Prior to the estimation of *Striga* base index value for each of the maize hybrids, the least square means for each of the traits were standardized to minimize the influence of the various scales employed for their measurement. The positive and negative index values implied that the hybrids were *Striga*-tolerant/resistant and *Striga*-susceptible, respectively [32].

Under *Striga*-free conditions, rank summation index (RSI) according to Ref. [33] was used to categorize the hybrids and the six checks in order of superiority. Total RSI values for plant height, plant aspect, days to 50 % silking, ears per plant, ear aspect, and grain yield (traits with significant variances) for the hybrids were used for the ordering of hybrids in this study. The lowest total rank value indicated the most superior hybrid while the highest total rank value showed the least performing hybrid. Correlation and regression analyses were carried out to study the relationships among the variables measured in the study using PROC CORR and PROC REG in SAS, respectively.

The average grain yield (t ha^−1^) of the eight most-superior hybrids, the three least-performing hybrids and the most-superior check identified using the *Striga* base index and rank summation index across different environments, were analyzed separately by GGE biplot to identify maize hybrids that combined stability with high grain yield across each environment. The average environment axis (AEA) was the line that passed through the average environment and the biplot origin with single arrow. Also, projections of hybrid markers to the AEA indicated the average yields of the hybrids [34]. Furthermore, the AEA ordinate, which was perpendicular to the AEA abscissa, approximated the genotype × environment interaction associated with each hybrid and was a measure of the instability of the hybrids across environments; the greater the projection onto the AEA ordinate, notwithstanding the direction, the more unstable was the hybrid [34]. GGE biplot model according to Ref. [35] and genotype × environment analysis with R for Windows (GEA-R) software version 4.0 [36] were used for the analysis.

## 3 Results

### 3.1 Analyses of variance for the agronomic traits of the 150 F_1_ maize hybrids in *Striga*-infested and *Striga*-free environments across two locations in Nigeria

The combined analysis of variance (ANOVA) for grain yield and other traits that were common to the seven (7) research environments in this study revealed that hybrid and environment main effects as well as their interaction (sources of variation) had significant (*P* < 0.001) mean squares for all measured traits except the hybrid mean squares for ear aspect and hybrid × environment mean squares for ear height (Table 1). The ANOVA across the four *Striga*-infested environments equally showed that the variances for all variables were significant (*P* < 0.001) for environment, hybrid and hybrid × environment components of variation except the environment and hybrid variances for plant aspect (Table 2). However, ANOVA across the three *Striga*-free environments, showed that the environment, hybrid and hybrid × environment interaction mean squares had significant (p < 0.001) variances for every attribute except hybrid × environment's variances for plant height and days to 50 % silking (Table 3).

**Table 1:** Mean squares extracted from the analysis of variance combined for grain yield and other measured traits of the 150 F_1_ hybrids and the checks evaluated across four *Striga*-infested and three *Striga*-free conditions at Mokwa and Abuja, Nigeria in 2016 and 2017.

Source of variation	Degree of freedom	Grain yield (kg ha^−1^)	Plant height (cm)	Number of ears per plant	Ear aspect (1–9)	Ear height (cm)	Days to 50 % silking (days)	Days to 50 % anthesis (days)	Anthesis-silking interval (days)
**Environment (E)**	6	880249441.00∗∗∗	33906.00∗∗∗	7.57∗∗∗	0.63∗∗∗	26048.00∗∗∗	1071.41∗∗∗	908.08∗∗∗	190.85∗∗∗
**Rep(E)**	7	8164563.00ns	382.14ns	0.11ns	0.03ns	404.79ns	6.22ns	4.71ns	3.36ns
**Block(E∗Rep)**	168	4097669.00∗∗∗	498.87∗∗∗	0.06∗∗∗	0.02∗∗∗	243.58∗∗∗	9.43∗∗∗	5.48∗∗∗	2.15∗∗∗
**Hybrid (H)**	155	2464272.00∗∗∗	414.99∗∗∗	0.05∗	0.01ns	172.77∗∗	32.66∗∗∗	31.08∗∗∗	3.65∗∗∗
**H∗E**	930	1505166.00∗∗∗	206.11∗∗	0.04∗∗∗	0.01∗∗∗	128.68ns	4.19∗∗∗	2.26∗∗∗	2.03∗∗∗
**Error**	917	947777.00	169.05	0.03	0.01	124.58	3.07	1.73	1.36

∗,∗∗,∗∗∗ = significant at p < 0.05, 0.01 and 0.001, respectively; ns = not significant.

**Table 2:** Trait mean performance and *Striga* base indices of eight best hybrids, the most superior checks and three worst hybrids of the 150 F_1_ hybrids evaluated under *Striga* infestation at Abuja and Mokwa, Nigeria.

HYBRID	aYIELD (t ha^−1^)	PLTH (cm)	EPP	EASP (1–9)	DYSK (days)	RAT1 (1–9)	RAT2 (1–9)	CO1	CO2	STRIGA-INDEX
LINE61 × LINE49	5.2	168.9	0.9	4	54	3	4	11	11	13.6
LINE61 × LINE44	4.4	163.7	0.8	4	55	3	4	5	5	11.2
LINE61 × LINE33	4.1	173.6	0.9	4	53	3	4	6	8	10.2
LINE52 × LINE49	4.4	167.3	0.8	4	54	4	4	7	7	9.6
LINE42 × LINE5	4.1	165.8	0.9	4	55	3	4	15	18	8.7
LINE44 × LINE53	4.0	176.3	0.9	4	56	3	4	9	9	8.6
LINE25 × LINE42	3.9	171.6	1.0	5	57	4	4	9	10	8.5
LINE62 × LINE33	4.1	181.9	0.8	4	54	3	4	13	13	8.5
TZEEI 79 × TZEEI 9	3.4	160.4	0.8	5	52	4	5	13	14	3.1
LINE30 × LINE62	2.5	165.1	0.5	6	56	5	5	23	22	−9.1
LINE32 × LINE61	2.1	150.3	0.6	5	54	5	6	20	21	−10.1
LINE54 × LINE5	2.4	163.4	0.6	6	54	5	6	24	30	−11.4
**Statistics**										
Mean	3.3	166.3	0.7	5	55	4	5	14	16	
Max	5.2	181.9	1.0	6	60	6	6	27	30	
Min	2.0	150.3	0.5	4	51	3	4	5	5	
Lsd (0.05)	2.0	25.1	0.4	2	4	2	2	16	17	
Probability of F for environment (E)	∗∗∗	∗∗∗	∗∗∗	ns	∗∗∗	∗∗	∗	∗∗	∗∗	
Probability of F for hybrid (H)	∗∗	∗∗	∗∗	ns	∗∗∗	∗∗∗	∗∗∗	∗∗∗	∗∗∗	
Probability of F for H × E	∗∗∗	∗∗	∗∗∗	∗∗∗	∗∗∗	∗∗∗	∗∗∗	∗	∗	

∗, ∗∗, ∗∗∗ and ns = significant at p < 0.05, p < 0.01, p < 0.001 and not significant, respectively.

a YIELD, grain yield; PLTH, plant height; EPP, ears per plant; EASP, ear aspect; DYSK, days to 50 % silking; RAT1, *Striga* damage rating at 8 weeks after planting; RAT2, *Striga* damage rating at 10 weeks after planting; CO1, *Striga* emergence count at 8 weeks after planting; CO2, *Striga* emergence count at 10 weeks after planting; *STRIGA*-INDEX, *Striga* base index.

**Table 3:** Grain yield and other characters of the eight best hybrids, the most superior check and the three least performing hybrids of the 150 F_1_ hybrids evaluated under *Striga*-free environments.

HYBRID	aYIELD (t ha^−1^)	PLTH (cm)	EPP	EASP (1–9)	DYSK (days)	PASP (1–9)	RSI
LINE53 × LINE 24	5.7	167.4	0.9	3	54	4	132
LINE53 × LINE 27	5.6	179.3	1.0	4	53	4	138
LINE49 × LINE 75	6.3	163.0	1.0	4	55	5	155
LINE56 × LINE 44	5.9	173.7	0.9	4	55	4	157
LINE53 × LINE 25	5.8	169.2	1.0	4	53	4	161
LINE55 × LINE 24	5.6	166.9	0.9	3	53	4	183
LINE53 × LINE 26	5.8	168.2	1.0	4	52	4	184
LINE44 × LINE 55	6.3	167.7	0.8	3	54	4	198
TZdEEI_1 × TZdEEI 9	3.8	164.5	0.7	5	54	5	585
LINE 30 × LINE11	3.6	143.3	0.8	5	51	6	848
LINE69 × LINE11	3.6	143.9	0.8	5	51	5	872
(TZEEI 82 × TZEEI 79) × TZEEI 95	3.4	140.3	0.8	5	48	5	877
**Statistics**							
Mean	4.9	159.0	0.9	4	53	5	
Max	6.3	179.3	1.0	6	57	6	
Min	3.1	134.7	0.6	3	48	4	
Lsd (0.05)	1.7	25.8	0.2	2	3	1	
Probability of F for environment (E)	∗∗∗	∗∗∗	∗∗∗	∗∗∗	∗∗∗	∗∗∗	
Probability of F for hybrid (H)	∗∗	∗∗∗	∗	∗∗∗	∗∗∗	∗∗	
Probability of F for H × E	∗∗∗	ns	∗∗	∗∗∗	ns	∗∗	

∗, ∗∗, ∗∗∗ and ns = significant at p < 0.05, p < 0.01, p < 0.001 and not significant, respectively.

a YIELD, grain yield; PLTH, plant height; EPP, ears per plant; EASP, ear aspect; DYSK, days to 50 % silking; PASP, plant aspect; RSI, rank summation index.

### 3.2 *Striga* base index, rank summation index and mean performance of the 150 F_1_ maize hybrids in *Striga*-infested and *Striga*-free environments

Across *Striga*-infested environments, *Striga* base index values varied from −11.4 for LINE 54 × LINE 5 to 13.6 for LINE 61 × LINE 49 (Table 2). Also, grain yield of the hybrids ranged from 2.0 to 5.2 t ha^−1^ with the average of 3.3 t ha^−1^. Compared to the performance under *Striga*-free environments, the mean grain yield reduction across *Striga*-infested environments was 33 %. The highest-yielding hybrid, LINE 61 × LINE 49 (5.2 t ha^−1^), although was not significantly different in grain yield when compared with other top-most yielding hybrids (identified through the *Striga* base index) as well as the most superior check (TZEEI 79 × TZEEI 9) under *Striga* infestation, it was statistically different from the three worst hybrids identified across the stress environments (Table 2).

Essentially, the eight superior provitamin A quality protein maize hybrids identified by the *Striga* base index (as *Striga* tolerant/resistant) were not statistically different from one another and the most superior check in respect of the traits assessed across *Striga*-infested environments. Nevertheless, significant differences existed between some of the top-most hybrids and the three worst hybrids (Table 2). For instance, the average performance of LINE 62 × LINE 33 with respect to plant height (181.9 cm) was significantly different from that of LINE 62 × LINE 33 (150.3 cm). In addition, significant differences were observed between LINE 25 × LINE 42 for ears per plant (1.0) and each of the worst-performing hybrids, with 0.5 and 0.6 for the same trait. Of the eight best hybrids, seven (with a *Striga* damage score of 4, for ear aspect) were significantly different from two of the worst hybrids (LINE 54 × LINE 5 and LINE 30 × LINE 62) with a score of 6 for ear aspect.

Across *Striga*-free environments on the other hand, rank summation index ranged from 132 for LINE 53 × LINE 24 to 877 for (TZEEI 82 × TZEEI 79) × TZEEI 95, a three-way-cross hybrid check (Table 3). Here, the eight top-most hybrids were significantly different from the most superior check (TZdEEI 1 × TZdEEI 9) and the three worst hybrids, with respect to plant height, ear aspect and grain yield (Table 3). Also, significant differences were observed among the eight hybrids and the check as well as the worst hybrids in terms of days to 50 % silking, plant aspect and ears per plant across the *Striga*-free environments. Grain yield was maximum (6.3 t ha^−1^) for the two hybrids, LINE4 4 × LINE 55 and LINE 49 × LINE 75, but was minimum (3.4 t ha^−1^) for (TZEEI 82 × TZEEI 79) × TZEEI 95 (Table 4). The two hybrids with the highest grain yield in the *Striga*-free environment performed significantly better than the check, TZdEEI 1 × TZdEEI 9 (3.8 t/ha) by 66 %.

**Table 4:** Coefficients of correlation (r) between each pair of traits of 150 F_1_ hybrids evaluated under *Striga* infestation (above diagonal) and *Striga*-free conditions (below diagonal).

	aYIELD	DYSK	EPP	PLHT	EASP	RAT1	RAT2	CO1	CO2
**YIELD**		−0.13ns	0.65∗∗∗	0.34∗∗∗	−0.84∗∗∗	−0.73∗∗∗	−0.77∗∗∗	−0.29∗∗∗	−0.28∗∗∗
**DYSK**	0.25∗∗		−0.28∗∗∗	−0.04ns	0.22∗∗	0.10ns	0.01ns	−0.29∗∗∗	−0.24∗∗
**EPP**	0.36∗∗∗	−0.21∗∗		0.16ns	−0.63∗∗∗	−0.63∗∗∗	−0.62∗∗∗	−0.16ns	−0.14ns
**PLHT**	0.49∗∗∗	0.11ns	0.21∗∗		−0.39∗∗∗	−0.38∗∗∗	−0.34∗∗∗	−0.22∗∗	−0.22∗∗
**EASP**	−0.72∗∗∗	−0.01ns	−0.35∗∗∗	−0.42∗∗∗		0.72∗∗∗	0.75∗∗∗	0.29∗∗∗	0.30∗∗∗
**RAT1**	–	–	–	–	–		0.90∗∗∗	0.28∗∗∗	0.27∗∗∗
**RAT2**	–	–	–	–	–	–		0.34∗∗∗	0.33∗∗∗
**CO1**	–	–	–	–	–	–	–		0.94∗∗∗
**CO2**	–	–	–	–	–	–	–	–	
**PASP**	−0.58∗∗∗	−0.10ns	−0.30∗∗∗	−0.45∗∗∗	0.61∗∗∗	–	–	–	–

∗, ∗∗, ∗∗∗ and ns = r is significant at p < 0.05, p < 0.01, p < 0.001 and not significant, respectively, for the hypothesis test Ho: ρ = 0.

a YIELD, grain yield; DYSK, days to 50 % silking; EPP, ears per plant; PLHT, plant height; EASP, ear aspect; RAT1, *Striga* damage rating at 8 weeks after planting; RAT2, *Striga* damage rating at 10 weeks after planting; CO1, *Striga* emergence count at 8 weeks after planting; CO2, *Striga* emergence count at 10 weeks after planting; PASP, plant aspect.

### 3.3 Relationships among the agronomic traits of the 150 F_1_ hybrids in *Striga*-infested and *Striga*-free environments

The results of correlation analysis showed that grain yield had significant and negative relationships with number of ears per plant (r = −0.7; *P* < 0.001), ear aspect (r = −0.8; *P* < 0.001) and *Striga* damage scores at 8 and 10 WAP (r > −0.7 in both cases; *P* < 0.001) under *Striga*-infested environment on one hand, and ear aspect (r = −0.7; *P* < 0.001) and plant aspect (r = −0.6; *P* < 0.001) under *Striga*-free conditions, on the other (Table 4).

**Fig. 1:**
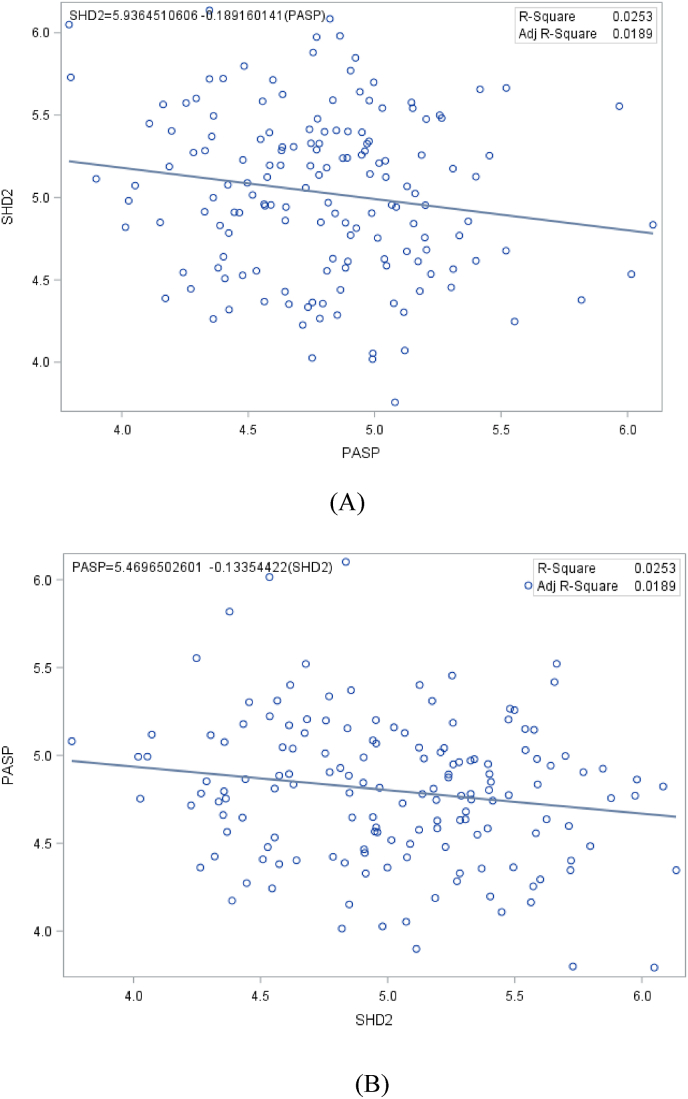
Regression of *Striga* host-damage score at 10 weeks after planting (SHD2) under *Striga* infestation on plant aspect (PASP) under *Striga*-free conditions and vice versa as revealed in (A) and (B), respectively.

In addition, stepwise regression analyses of grain yield on other important agronomic variables of the maize hybrids across the research environments revealed that *Striga* damage score at 10 WAP (59 %), ears per plant (5 %)) and plant height (1 %) were largely responsible for the variation in grain yield under *Striga*-infested environments while plant aspect (34 %), plant height (7 %), ears per plant (5 %) and days to 50 % silking (3 %) were the significant contributors to the differences observed in grain yield under *Striga*-free conditions (Table 5). In addition, regression of *Striga* damage score at 10 WAP (a character phenotyped and ranked under *Striga*-infested environment) on plant aspect (under *Striga*-free conditions) and vice versa showed a negative and weak relationship between the traits (Fig. 1).

**Table 5:** Stepwise regression of grain yield on important agronomic traits of 150 F_1_ maize hybrids under *Striga*-infested and *Striga*-free environments.

Environment	Traits	Partial R-square	Probability > F
*Striga*-Infested	Striga host-damage score at 10 weeks after planting	0.59	<0.0001
	Ears per plant	0.05	<0.0001
	Plant height	0.01	0.0354

*Striga*-free	Plant aspect	0.34	<0.0001
	Plant height	0.07	<0.0001
	Ears per plant	0.05	0.0003
	Days to 50 % silking	0.03	0.007

**Fig. 2:**
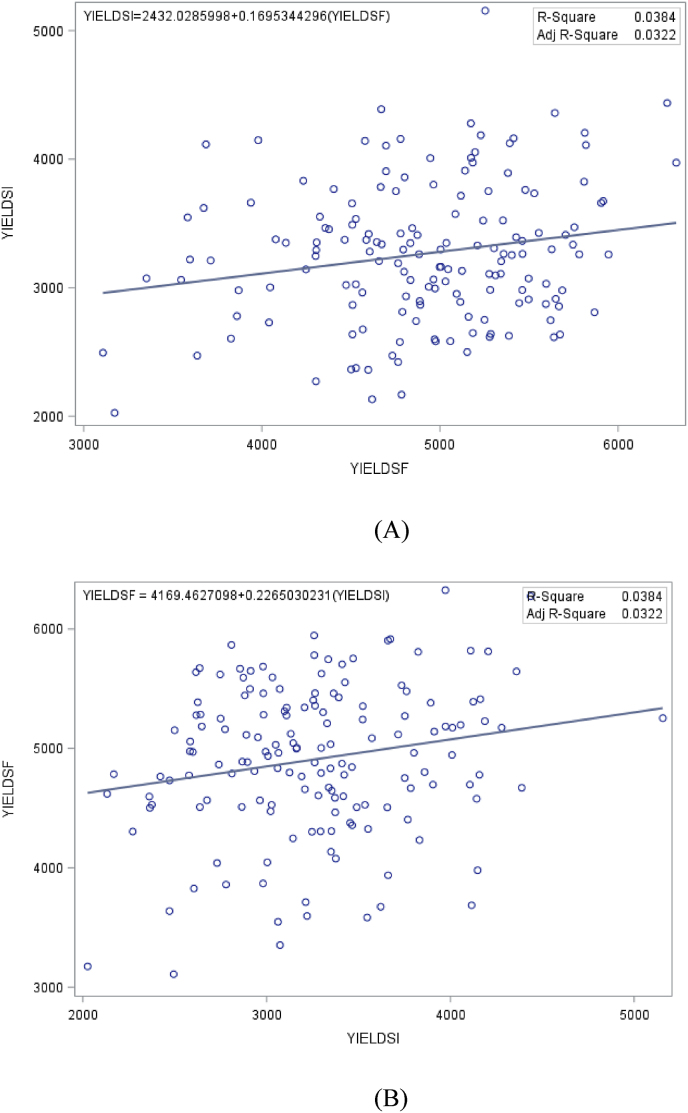
Regression of grain yield under *Striga*-infestation (YIELDSI) on grain yield under *Striga*-free (YIELDSF) and vice versa as revealed in (A) and (B), respectively.

The regression slopes and R^2^ values in both cases were less than 20 % and 3 %, respectively. Similarly, the regression of yield under *Striga*-infested conditions on the grain yield under *Striga*-free environments and vice versa revealed a positive and weak associations between the traits (regression slopes <25 % in both cases; R^2^ = 3.8 %) (Fig. 2).

### 3.4 Grain yield and stability of selected single-cross maize hybrids across *Striga*-infested and *Striga*-free environments across the two locations in Nigeria

**Fig. 3:**
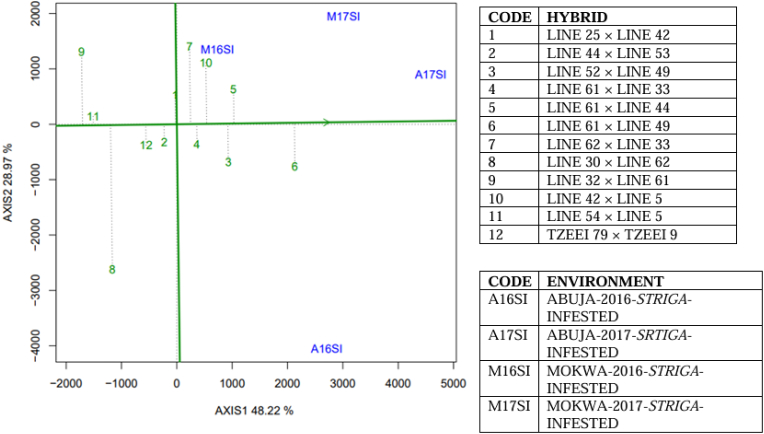
The “mean versus stability” view of the GGE biplot of the eight best and three worst hybrids including the most superior check identified by the *Striga* base index across four *Striga*-infested environments in Nigeria.

**Fig. 4:**
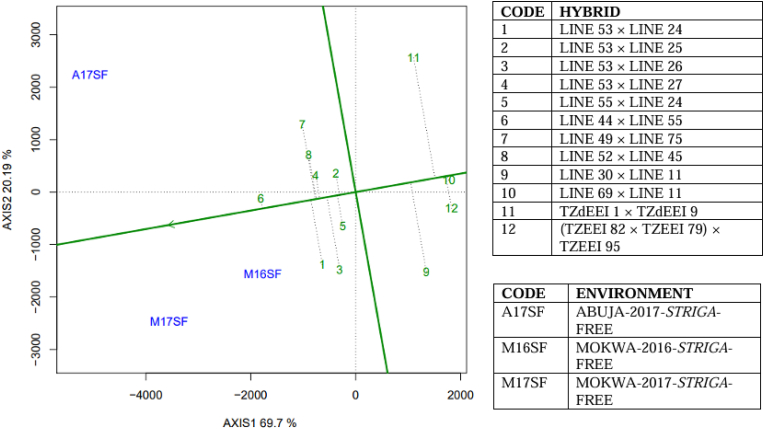
The “mean versus stability” view of the GGE biplot of the eight best and three worst hybrids including the most superior check identified by rank summation index across three *Striga*-free environments in Nigeria.

Average yield and stability of twelve selected hybrids (eight best, three worst and the best check) in the two contrasting *Striga* environments are presented in Fig. 3, Fig. 4. Across *Striga*-infested environments, 77 % (PC1 = 48 % and PC2 = 29 %) of the variability in the mean grain yield of the hybrids was explained by the two principal component axes (Fig. 3), while under *Striga*-free environments, the two axes explained 90 % (PC1 = 70 % and PC2 = 20 %) of the difference in grain yield (Fig. 4). Across *Striga*-infested environments, Hybrid 6 (LINE 61 × LINE 49) and Hybrid 9 (LINE 32 × LINE 61) had the longest and shortest projections on the AEA, respectively (Fig. 3), indicating that they were the highest-yielding and lowest-yielding hybrids in the environments. However, across *Striga*-free environments, Hybrid 6 (LINE 44 × LINE 55) had the longest projection onto the AEA while Hybrid 10 (LINE 69 × LINE 11) had the shortest projection.

Under *Striga*-infested conditions, Hybrids 2 (LINE 44 × LINE 53), 4 (LINE 61 × LINE33), 11 (LINE 54 × LINE 5) and 12 (TZEEI 79 × TZEEI 9) had shorter projections onto the AEA ordinate across *Striga*-infested environments and were the most stable; however, the average yield performance of the three of them (Hybrids 2, 11 and 12) were lower than the mean performance of all the hybrids across the environments. Therefore, the hybrids: 6 (LINE 61 × LINE 49).

SHD10, *Striga* damage rating at 10 weeks after planting; EPP, number of ears per plant; PLHT, Plant height (cm); PASP, Plant aspect; DYSK, Days to 50 % silking.

5 (LINE 61 × LINE 44) and 3 (LINE 52 × LINE 49) combined high mean performance and grain yield stability under artificial infestation with *Striga* seeds – the hybrids had relatively shorter projections onto the AEA ordinate. Across *Striga*-free environments, Hybrid 6 (LINE 44 × LINE 55) had the longest projection on the AEA abscissa and shortest projection on the AEA ordinate, suggesting that it combined high grain yield performance with high stability. However, Hybrid 11 (TZdEEI 1 × TZdEEI 9) had lower projection on the AEA abscissa and longest projection onto the AEA ordinate, indicating that it combined low yield performance with excessive instability.

## 4 Discussion

Genotype and environment as well as their interaction play a significant role in crop performance. Adetimirin, Aken’Ova and Kim [9] reported that plant/crop productivity is influenced by biotic stresses. Crop productivity is in turn influenced by soil fertility/productivity and can be restored through fallowing [47]. *Striga hermonthica* infestation is a significant biotic stress of maize, particularly in soil with poor fertility, and can cause 100 % grain yield loss in critical situation [4]. Thus, diverse strategies have been recommended to control the negative effects of *Striga* damage on maize yield and production [12,14]. The significant variances obtained for environment and hybrid sources of variation in the combined ANOVA, for majority of the traits studied across environments indicated that there were significant variations among the maize hybrids for studied traits and that the environments were distinct in discriminating among the maize hybrids. This is a strong indication that selection is possible for improvement of the measured traits. The uniqueness of the environments used in this study might be attributed to the variation due to types of soil, rainfall patterns and *Striga hermonthica* biotypes in the research sites [31,37]. This justified why evaluations under artificial *Striga* infestation are usually carried out at different locations in the southern Guinea savanna agro-ecological zones of Nigeria where *Striga* is a serious threat to maize production [25].

The significant hybrid × environment variances obtained among the maize hybrids for the measured variables/characters examined under *Striga* infestation and for majority of the traits studied under *Striga*-free conditions showed the significant effect of the environment on the performance of the characters. In other words, the performances of the hybrids with respect to those traits were inconsistent across the environments – an indication of significant genotype-environment interaction. Similar results were obtained by Ref. [31] when maize hybrids with *Zea diploperennis* genes were evaluated under contrasting *Striga* environments. Also, the result agreed with the report of [9].

The lack of significant differences observed among the eight most superior (*Striga*-tolerant/resistant) biofortified extra-early maize hybrids and the most superior check in the study suggests that the hybrids and the check were comparable in their performances with respect to the agronomic traits across the *Striga*-infested test environments. However, significant differences were observed across *Striga*-free environments among the eight top-most hybrids and the most superior check for grain yield, plant height and plant aspect, indicating that the hybrids and the check were not similar in performance for the traits. The differential performance of the maize hybrids evaluated in this study across environments suggested that the environments had differential effects on the performance of the hybrids. Eze et al. [38] reported that it is typical for different maize genotypes to display varying performance under contrasting environmental conditions. Aboyeji and Haruna [46] also observed significant difference even in the performance of a maize genotype evaluated under different environmental conditions in Sudan Savanna of Nigeria. In the same vein [48], reported a differential yield performance of a maize variety evaluated under varying soil nitrogen environments (split applications of urea) in Southwest Nigeria.

The *Striga* base index value 13.6 observed for LINE 61 × LINE 49 revealed the hybrid as the most *Striga*-tolerant/resistant in this study. *Striga* base index has been used to identify *Striga*-tolerant/resistant maize genotypes in previous studies [31,32,39]. The differences in performance of eight top-yielding hybrids and the checks as well as the dissimilarity of performances observed across each of the two sets of environments might be attributed to the different base indices used for the selection of the top-performing hybrids across the *Striga*-infested and the *Striga*-free test environments. This result is in consonance with the report of [40] who observed significant difference in genotype performance due to use of different indices for selection of genotypes.

The strong and significant associations observed between yield and the two agronomic characters (ear aspect and plant aspect) across *Striga*-free environments, and between yield and *Striga* damage scores as well as ear aspect across *Striga*-infested environments, suggested that those two traits (across each environment) showed similar associations with grain yield. *Striga* damage rating and ear aspect were two of the traits with strong association with grain yield under *Striga* infestation [4]. While *Striga* damage score at 10 WAP made the highest contribution to the difference in grain yield under *Striga* infestation, plant aspect accounted for the greatest variation in grain yield in *Striga*-free environments, suggesting that grain yield performance across each of the environments could be predicted, by those traits to a great extent. *Striga* damage rating and ear aspect are two of the three most reliable secondary traits identified for improving grain yield under *Striga*-infested environments [41].

The low regression coefficients and R-square values obtained when *Striga*-host damage score was regressed on plant aspect and vice versa, indicated that plant aspect under *Striga*-free conditions had no reliable effect on *Striga*-host damage rating and cannot be used to predict *Striga*-host damage score under *Striga*-infested environment. The similarity of the results obtained when grain yield across *Striga*-infested environments was regressed on grain yield under *Striga*-free environments and vice versa, showed that maize grain yield performance across one set of environments are unique and that grain yield performance in one environment might not be a good predictor for grain yield performance in another. These results justify the need to religiously phenotype a new set of maize varieties/hybrids, separately, under *Striga*-infested and *Striga*-free conditions in an effort to identify a high-yielding, *Striga*-resistant/tolerant maize variety. This result is contradictory to the earlier report by Ref. [42] who obtained 70 % R-squared value for regressing grain yield under stress on grain yield under stress-free condition and vice versa, and concluded that maize cultivars selected under stress might also have superior performance under stress-free conditions.

The main effect of GGE is a focal source of variation in the evaluation of genotypes under multi-environment trials and “mean vs stability” performance for genotype assessment across environments. It is one of the three important components that can be explained with GGE biplot [43]. Results of the GGE biplot analyses of the maize hybrids evaluated across the environments revealed that the principal component axes 1 and 2 explained more than 76 % of the total variations in grain yield, indicating that large proportion of the variations in grain yield were captured by the principal component axes and that the “mean vs stability” view of the GGE biplot should give reliable results. Across *Striga*-infested environments, Hybrid 6 (LINE 61 × LINE 49) combined the highest projection on AEA abscissa and relatively short projection on the AEA ordinate, indicating that the hybrid combined good mean performance and grain yield stability across *Striga*-infested environments. On the other hand, Hybrid 6 (LINE 44 × LINE 55) of the *Striga*-free environment displayed the longest projection on the AEA abscissa and shortest projection on the AEA ordinate. This implied that the hybrid gave maximum yield and stability across *Striga*-free environments. Different authors had earlier used the GGE biplot analysis to identify effectively stable maize genotypes in various studies [18,22,38,44].

## 5 Conclusions

Environment had significant effect on the average performance of the biofortified maize hybrids evaluated in this study; the average grain yield of the maize hybrids were 3.3 and 4.9 t ha^−1^ in *Striga*-infested and *Striga*-free environments, respectively. Of the 150 experimental maize hybrids studied, LINE 61 × LINE 44 and LINE 61 × LINE 49 were the most *Striga*-resistant/tolerant and could be used in either integrated *Striga* management or exploited in future breeding programmes for the development of superior genotypes. Plant aspect under *Striga*-free environments could not be used to predict *Striga* tolerance under *Striga*-infested conditions and vice versa and grain yield under *Striga*-free environment could not be used to predict grain yield under *Striga*-infested environment and vice versa. LINE 61 × LINE 49 and LINE 44 × LINE 55 were the highest-yielding and most stable hybrids identified in *Striga*-infested and *Striga*-free environments, respectively. These hybrids are recommended for on-farm evaluation and subsequent release to maize growers in areas affected by *Striga hermonthica* to boost maize production and contribute to food security in Africa.

## CRediT authorship contribution statement

**Solomon Adeyemi Oyekale:** Writing – original draft, Software, Investigation, Formal analysis, Conceptualization, Writing – review & editing, Visualization, Methodology, Funding acquisition, Data curation. **Baffour Badu-Apraku:** Validation, Resources, Methodology, Funding acquisition, Conceptualization, Writing – review & editing, Supervision, Project administration, Investigation, Data curation.

## Consent to participate

Not applicable.

## Consent to publish

All authors read and approved the publishing of this article.

## Ethical approval

Not applicable.

## Data availability statement

The datasets analyzed in this study and the materials used are available at the IITA CKAN repository.

## Funding

The 10.13039/501100011951African Union provided funding support for the PhD thesis research of the first author at the 10.13039/501100022496Pan African University Institute of Life and Earth Sciences (Including Health and Agriculture), 10.13039/501100004918University of Ibadan, Ibadan, Nigeria. Also, the Drought Tolerant Maize for Africa Project of the Bill and Melinda Gates Project (OPP1134248) provided funding support for the PhD thesis. However, the funding bodies did not participate in the design, collection, analysis, interpretation of data, and writing of the manuscript.

## Declaration of competing interest

The authors have nothing to declare.
