# Supplementary material for: Performance prediction and stability of maize hybrids in contrasting Striga environments
Source: J Agric Food Res. 2025 Dec;24:102405. doi: 10.1016/j.jafr.2025.102405 (PMC12679918; doi:10.1016/j.jafr.2025.102405)
Supplement: Multimedia component 1 [file mmc1.docx]

**Supplemental Table 1.** Designation, pedigree information and reactions of the inbred lines used for the study

| S/N | Designation | Pedigree Information | Reaction to *Striga* |
| --- | --- | --- | --- |
| 1 | ^¶^LINE 5 | 2009 TZEE-OR2 STR QPM S6 19-1/2-2/2-2/2-1/2-1/1 | Tolerant |
| 2 | LINE 11 | 2009 TZEE-OR2 STR QPM S6 20-2/2-3/3-1/2-1/2-1/1 | Susceptible |
| 3 | LINE 24 | 2009 TZEE-OR2 STR QPM S6 21-2/6-1/3-1/2-1/2-1/1 | Susceptible |
| 4 | LINE 25 | 2009 TZEE-OR2 STR QPM S6 21-2/6-1/3-2/2-1/2-1/2 | Tolerant |
| 5 | LINE 26 | 2009 TZEE-OR2 STR QPM S6 21-2/6-1/3-2/2-2/2-2/2 | Tolerant |
| 6 | LINE 27 | 2009 TZEE-OR2 STR QPM S6 21-2/6-2/3-2/2-2/2-2/2 | Tolerant |
| 7 | LINE 28 | 2009 TZEE-OR2 STR QPM S6 21-2/6-3/3-2/3-1/3-1/2 | Tolerant |
| 8 | LINE 29 | 2009 TZEE-OR2 STR QPM S5 21-2/6-3/3-2/3-2/3-2/2 | Susceptible |
| 9 | LINE 30 | 2009 TZEE-OR2 STR QPM S6 21-2/6-3/3-3/3-1/3-1/2 | Tolerant |
| 10 | LINE 32 | 2009 TZEE-OR2 STR QPM S6 21-5/6-1/2-1/2-3/3-1/1 | Tolerant |
| 11 | LINE 33 | 2009 TZEE-OR2 STR QPM S6 22-1/3-1/2-2/4-2/2-1/1 | Susceptible |
| 12 | LINE 35 | 2009 TZEE-OR2 STR QPM S6 22-1/3-1/2-3/4-1/2-1/1 | Susceptible |
| 13 | LINE 41 | 2009 TZEE-OR2 STR QPM S6 22-3/3-1/3-2/3-3/4-1/1 | Tolerant |
| 14 | LINE 42 | 2009 TZEE-OR2 STR QPM S6 22-3/3-2/3-3/3-1/3-1/2 | Tolerant |
| 15 | LINE 43 | 2009 TZEE-OR2 STR QPM S6 22-3/3-2/3-3/3-2/3-2/2 | Tolerant |
| 16 | LINE 44 | 2009 TZEE-OR2 STR QPM S6 22-3/3-3/3-1/3-1/3-1/3 | Tolerant |
| 17 | LINE 45 | 2009 TZEE-OR2 STR QPM S6 22-3/3-3/3-1/3-2/3-2/3 | Tolerant |
| 18 | LINE 49 | 2009 TZEE-OR2 STR QPM S6 22-3/3-3/3-2/2-1/1-1/1 | Tolerant |
| 19 | LINE 52 | 2009 TZEE-OR2 STR QPM S6 27-1/5-2/3-2/2-2/3-1/1 | Tolerant |
| 20 | LINE 53 | 2009 TZEE-OR2 STR QPM S6 27-1/5-2/3-1/2-3/3-1/1 | Susceptible |
| 21 | LINE 54 | 2009 TZEE-OR2 STR QPM S6 27-1/5-3/3-1/3-2/2-1/1 | Tolerant |
| 22 | LINE 55 | 2009 TZEE-OR2 STR QPM S6 27-1/5-3/3-2/3-1/2-1/2 | Susceptible |
| 23 | LINE 56 | 2009 TZEE-OR2 STR QPM S6 27-1/5-3/3-2/3-2/2-2/2 | Tolerant |
| 24 | LINE 57 | 2009 TZEE-OR2 STR QPM S6 27-1/5-3/3-3/3-1/1-1/1 | Tolerant |
| 25 | LINE 61 | 2009 TZEE-OR2 STR QPM S6 27-5/5-1/2-1/3-2/3-1/1 | Tolerant |
| 26 | LINE 62 | 2009 TZEE-OR2 STR QPM S6 27-5/5-1/2-3/3-2/3-1/1 | Tolerant |
| 27 | LINE 64 | 2009 TZEE-OR2 STR QPM S6 27-5/5-2/2-1/2-2/2-2/2 | Tolerant |
| 28 | LINE 69 | 2009 TZEE-OR2 STR QPM S6 34-1/1-3/3-1/1-2/4-1/2 | Tolerant |
| 29 | LINE 75 | 2009 TZEE-OR2 STR QPM S6 82-2/2-2/2-1/4-1/3-1/2 | Tolerant |
| 30 | LINE 76 | 2009 TZEE-OR2 STR QPM S6 82-2/2-2/2-1/4-3/3-2/2 | Tolerant |

^¶^LINE: Tropical *Zea* Extra-Early Provitamin A Quality Protein Maize Inbred Line (TZEEIORQ)

**Supplemental Table 2.** Characteristics of the 150 F_1_ hybrids and the checks used for the study.

| S/N | Hybrid | Attributes of the inbred lines in crosses | S/N | Hybrid | Attributes of the inbred lines in crosses |
| --- | --- | --- | --- | --- | --- |
| 1 | LINE 61 × LINE 49 | ^*^TT | 44 | LINE 69 × LINE 52 | TT |
| 2 | LINE 61 × LINE 44 | TT | 45 | LINE 49 × LINE 76 | TT |
| 3 | LINE 52 × LINE 49 | TT | 46 | LINE 44 × LINE 57 | TT |
| 4 | LINE 42 × LINE 5 | TT | 47 | LINE 75 × LINE 27 | TT |
| 5 | LINE 25 × LINE 42 | TT | 48 | LINE 45 × LINE 76 | TT |
| 6 | LINE 49 × LINE 75 | TT | 49 | LINE 28 × LINE 56 | TT |
| 7 | LINE 61 × LINE 43 | TT | 50 | LINE 26 × LINE 42 | TT |
| 8 | LINE 52 × LINE 45 | TT | 51 | LINE 76 × LINE 26 | TT |
| 9 | LINE 25 × LINE 64 | TT | 52 | LINE 5 × LINE 56 | TT |
| 10 | LINE 26 × LINE 41 | TT | 53 | LINE 64 × LINE 5 | TT |
| 11 | LINE 69 × LINE 62 | TT | 54 | LINE 26 × LINE 64 | TT |
| 12 | LINE 62 × LINE 45 | TT | 55 | LINE 75 × LINE 26 | TT |
| 13 | LINE 27 × LINE 42 | TT | 56 | LINE 45 × LINE 57 | TT |
| 14 | LINE 62 × LINE 43 | TT | 57 | LINE 43 × LINE 76 | TT |
| 15 | LINE 56 × LINE 44 | TT | 58 | LINE 5 × LINE 52 | TT |
| 16 | LINE 27 × LINE 64 | TT | 59 | LINE 45 × LINE 55 | TT |
| 17 | LINE 42 × LINE 30 | TT | 60 | LINE 76 × LINE 27 | TT |
| 18 | LINE 56 × LINE 43 | TT | 61 | LINE 56 × LINE 45 | TT |
| 19 | LINE 41 × LINE 30 | TT | 62 | LINE 5 × LINE 62 | TT |
| 20 | LINE 42 × LINE 69 | TT | 63 | LINE 41 × LINE 28 | TT |
| 21 | LINE 41 × LINE 69 | TT | 64 | LINE 52 × LINE 44 | TT |
| 22 | LINE 49 × LINE 55 | TT | 65 | LINE 44 × LINE 75 | TT |
| 23 | LINE 62 × LINE 44 | TT | 66 | LINE 5 × LINE 61 | TT |
| 24 | LINE 30 × LINE 61 | TT | 67 | LINE 28 × LINE 61 | TT |
| 25 | LINE 56 × LINE 49 | TT | 68 | LINE 43 × LINE 75 | TT |
| 26 | LINE 28 × LINE 62 | TT | 69 | LINE 32 × LINE 52 | TT |
| 27 | LINE 41 × LINE 32 | TT | 70 | LINE 28 × LINE 52 | TT |
| 28 | LINE 44 × LINE 76 | TT | 71 | LINE 42 × LINE 32 | TT |
| 29 | LINE 26 × LINE 54 | TT | 72 | LINE 64 × LINE 32 | TT |
| 30 | LINE 55 × LINE 26 | TT | 73 | LINE 64 × LINE 30 | TT |
| 31 | LINE 55 × LINE 25 | TT | 74 | LINE 49 × LINE 57 | TT |
| 32 | LINE 69 × LINE 61 | TT | 75 | LINE 57 × LINE 27 | TT |
| 33 | LINE 32 × LINE 62 | TT | 76 | LINE 30 × LINE 56 | TT |
| 34 | LINE 57 × LINE 25 | TT | 77 | LINE 25 × LINE 54 | TT |
| 35 | LINE 25 × LINE 41 | TT | 78 | LINE 41 × LINE 5 | TT |
| 36 | LINE 61 × LINE 45 | TT | 79 | LINE 54 × LINE 28 | TT |
| 37 | LINE 32 × LINE 56 | TT | 80 | LINE 64 × LINE 69 | TT |
| 38 | LINE 43 × LINE 55 | TT | 81 | LINE 64 × LINE 28 | TT |
| 39 | LINE 27 × LINE 41 | TT | 82 | LINE 75 × LINE 25 | TT |
| 40 | LINE 52 × LINE 43 | TT | 83 | LINE 57 × LINE 26 | TT |
| 41 | LINE 62 × LINE 49 | TT | 84 | LINE 54 × LINE 69 | TT |
| 42 | LINE 69 × LINE 56 | TT | 85 | LINE 54 × LINE 32 | TT |
| 43 | LINE 42 × LINE 28 | TT | 86 | LINE 45 × LINE 75 | TT |

**Supplemental Table 2.** Continued.

| S/N | Hybrid | Attributes of the inbred lines in crosses | S/N | Hybrid | Attributes of the inbred lines in crosses |
| --- | --- | --- | --- | --- | --- |
| 87 | LINE 43 × LINE 57 | TT | 124 | LINE 35 × LINE 32 | ST |
| 88 | LINE 55 × LINE 27 | TT | 125 | LINE 35 × LINE 5 | ST |
| 89 | LINE 27 × LINE 54 | TT | 126 | LINE 29 × LINE 42 | ST |
| 90 | LINE 54 × LINE 30 | TT | 127 | LINE 11 × LINE 44 | ST |
| 91 | LINE 76 × LINE 25 | TT | 128 | LINE 35 × LINE 28 | ST |
| 92 | LINE 30 × LINE 52 | TT | 129 | LINE 35 × LINE 30 | ST |
| 93 | LINE 30 × LINE 62 | TT | 130 | LINE 33 × LINE 57 | ST |
| 94 | LINE 54 × LINE 5 | TT | 131 | LINE 11 × LINE 43 | ST |
| 95 | LINE 61 × LINE 33 | ^¶^TS | 132 | LINE 53 × LINE 26 | ST |
| 96 | LINE 44 × LINE 53 | TS | 133 | LINE 29 × LINE 64 | ST |
| 97 | LINE 62 × LINE 33 | TS | 134 | LINE 53 × LINE 25 | ST |
| 98 | LINE 56 × LINE 33 | TS | 135 | LINE 11 × LINE 45 | ST |
| 99 | LINE 44 × LINE 55 | TS | 136 | LINE 29 × LINE 41 | ST |
| 100 | LINE 27 × LINE 35 | TS | 137 | LINE 24 × LINE 41 | ST |
| 101 | LINE 43 × LINE 53 | TS | 138 | LINE 24 × LINE 64 | ST |
| 102 | LINE 69 × LINE 11 | TS | 139 | LINE 24 × LINE 42 | ST |
| 103 | LINE 26 × LINE 35 | TS | 140 | LINE 53 × LINE 27 | ST |
| 104 | LINE 45 × LINE 53 | TS | 141 | LINE 29 × LINE 54 | ST |
| 105 | LINE 76 × LINE 29 | TS | 142 | LINE 24 × LINE 54 | ST |
| 106 | LINE 49 × LINE 53 | TS | 143 | LINE 32 × LINE 61 | ST |
| 107 | LINE 25 × LINE 35 | TS | 144 | LINE 33 × LINE 55 | ^‡^SS |
| 108 | LINE 52 × LINE 33 | TS | 145 | LINE 24 × LINE 35 | SS |
| 109 | LINE 55 × LINE 29 | TS | 146 | LINE 33 × LINE 53 | SS |
| 110 | LINE 28 × LINE 11 | TS | 147 | LINE 11 × LINE 33 | SS |
| 111 | LINE 57 × LINE 29 | TS | 148 | LINE 53 × LINE 29 | SS |
| 112 | LINE 5 × LINE 11 | TS | 149 | LINE 29 × LINE 35 | SS |
| 113 | LINE 57 × LINE 24 | TS | 150 | LINE 53 × LINE 24 | SS |
| 114 | LINE 75 × LINE 24 | TS | 151 | TZEEI 79 × TZEEI 9 | ^a^CHECK 1 |
| 115 | LINE 75 × LINE 29 | TS | 152 | TZdEEI 1 × TZdEEI 9 | CHECK 2 |
| 116 | LINE 55 × LINE 24 | TS | 153 | TZdEEI 9 × TZdEEI 12 | CHECK 3 |
| 117 | LINE 32 × LINE 11 | TS | 153 | (TZEEI 82 × TZEEI 79) × TZEEI 95 | ^b^CHECK 4 |
| 118 | LINE 76 × LINE 24 | TS | 155 | TZEE-Y Pop STR C5 × TZEEI 82 | CHECK 5 |
| 119 | LINE 30 × LINE 11 | TS | 156 | TZEE-Y Pop STR C5 × TZEEI 58 | CHECK 6 |
| 120 | LINE 33 × LINE 75 | ^#^ST |  |  |  |
| 121 | LINE 11 × LINE 49 | ST |  |  |  |
| 122 | LINE 35 × LINE 69 | ST |  |  |  |
| 123 | LINE 33 × LINE 76 | ST |  |  |  |

^*^TT: Tolerant × Tolerant; ^¶^TS: Tolerant × Susceptible; ^#^ST: Susceptible × Tolerant; ^‡^SS: Susceptible × Susceptible; ^a^Checks 1-3 are tolerant to *Striga* while ^b^Checks 4-6 are susceptible.
